# Simultaneous Laparoscopic Surgery for Bladder Diverticulum and Urachal Remnant: A Case Report

**DOI:** 10.1155/2019/5785189

**Published:** 2019-08-28

**Authors:** Masaki Murata, Kohei Inui, Moto Hasegawa, Yohei Ikeda, Yuki Nakagawa, Noboru Hara, Tsutomu Nishiyama

**Affiliations:** ^1^Department of Urology, Uonuma Institute of Community Medicine, Niigata University Medical and Dental Hospital, Niigata, Japan; ^2^Department of Diagnostic Radiology, Uonuma Institute of Community Medicine, Niigata University Medical and Dental Hospital, Niigata, Japan

## Abstract

A 44-year-old woman visited a hospital with microscopic hematuria in June 2009. Computed tomography showed a large bladder diverticulum at right lateral wall and urachal remnant. Cystoscopic examination showed a large diverticulum at the right lateral wall that closes to the dome. She was followed up closely without therapeutic intervention; however, the residual urine increased gradually and frequent bladder diverticulitis developed. She underwent laparoscopic bladder diverticulectomy and excision of the urachal remnant simultaneously without any complications in August 2017. Laparoscopic approach for a large bladder diverticulum and urachal remnant is useful with safe, effective, and minimally invasive.

## 1. Introduction

Bladder diverticulum is a bladder abnormality that mainly acquired, forms by the herniation of bladder mucosa through the muscle layers [1]. It can cause lower urinary tract symptoms (LUTS), urinary tract infections (UTIs), residual urine, bladder stones, and malignancy [1]. Urachal remnant is a rare congenital anomaly that is caused by a failure in the obliteration of the allantois [2]. A surgical approach for these diseases is not necessary in many cases. On the other hand, surgical treatment is performed when symptomatic or malignant [1–4]. Recently, a minimally invasive procedure is required, and the laparoscopic approach is useful in this regard [3]. We report a laparoscopic surgery for a patient with simultaneous occurrence of bladder diverticulum and urachal remnant.

## 2. A Case Report

**Figure 1:**
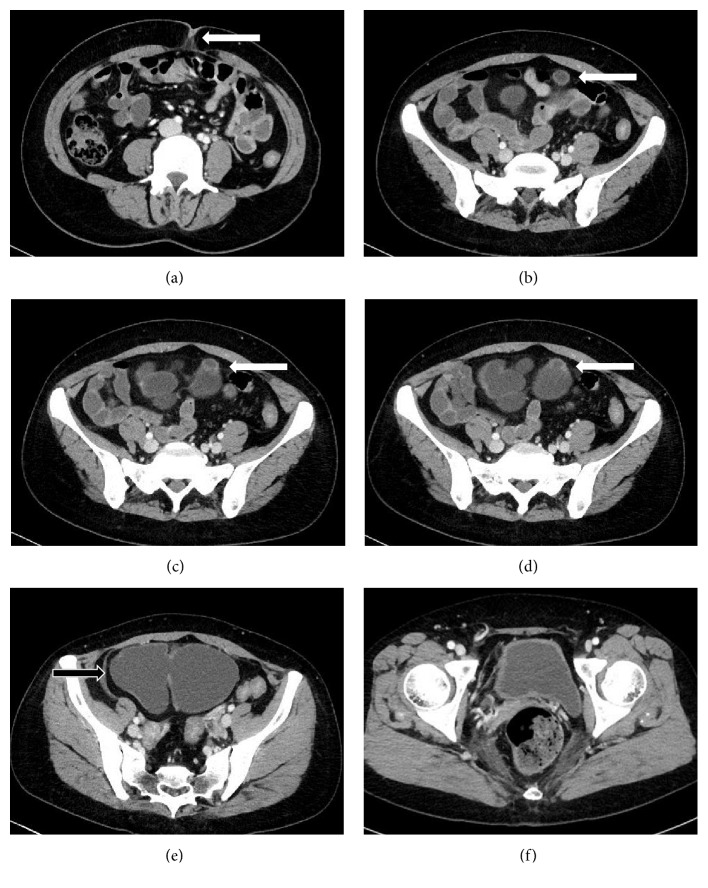
Abdominal CT shows urachal remnant and bladder diverticulum. (a–d) The fibrous cord runs from the umbilicus to the dome of the bladder (arrow). (e) A large bladder diverticulum locates in the right lateral wall (arrow). (f) CT findings nine months after the operation revealing no recurrence of the diverticula.

**Figure 2:**
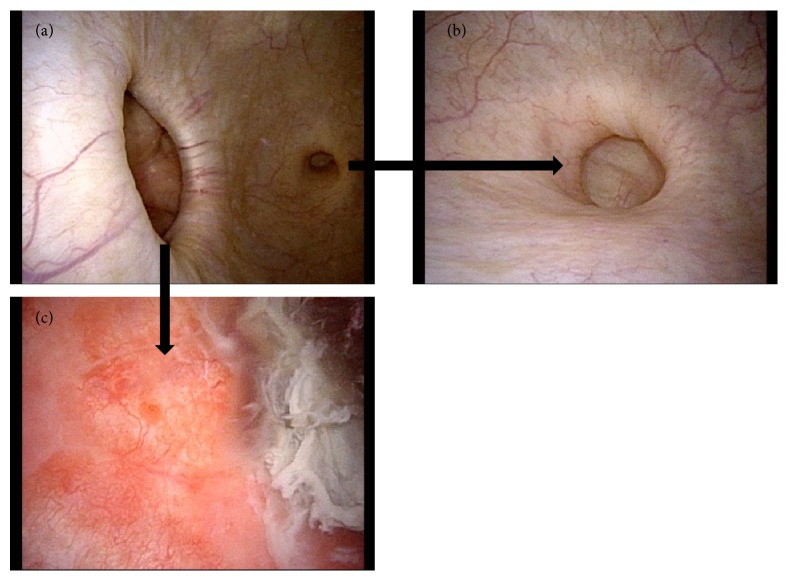
Cystoscopic examination showed a diverticulum neck at the right wall and urachal diverticulum at the dome of bladder. (a, b) The diverticular mucosa was redness, and (c) erosion.

A 44-year-old woman visited a nearby hospital with microscopic hematuria in June 2009. Her past medical history is tetralogy of Fallot. She had no medications at the time of the visit. There was no abnormality on physical examination. Computed tomography (CT) showed urachal remnant (urachal diverticulum at the dome of the bladder) and a large bladder diverticulum at the right lateral wall (Figures 1(a)–1(e)). Urine cytology was negative. Since she did not have any symptoms, she was followed up without therapeutic intervention. However, the residual urine had been increasing gradually, and she was referred to our hospital for continuous follow-up in February 2016. The postvoid residual urine was 150 ml at the time. After six months, she complained of severe dysuria and repeated urinary tract infection possibly caused by bladder diverticulitis. Cystoscopic examination showed a large diverticulum at the right wall, and diverticular mucosa was reddish and erosive (Figures 2(a) and 2(c)). Urachal diverticulum was small and without inflammation (Figure 2(b)). Antibiotic therapy with Levofloxacin was effective for UTI. She was started treatment with Urapidil of 30 mg/day simultaneously; however, the postvoid residual urine and dysuria did not improve.

**Figure 3:**
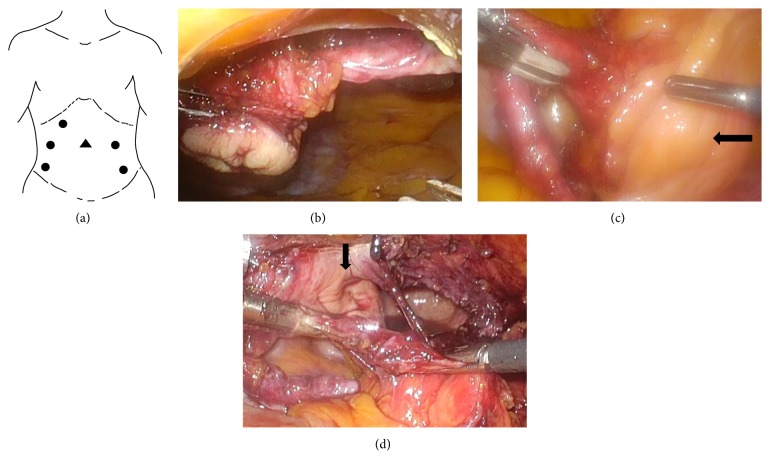
(a) The placements of the trocar port. ●: operator's and assistant's port; ▲: camera port. (b) The umbilicus and umbilical ligament viewed from the right working port. (c) Excision of the umbilical ligament just above the bladder dome. The bulging of the bladder diverticulum is identified just to the right side of the bladder dome (arrow). (d) After resection of the bladder diverticulum. The urachal diverticulum is identified just to the left of the resection site (arrow).

We performed bladder diverticulectomy and excision of urachal remnant simultaneously with the laparoscopic approach in August 2017. We cut out the umbilicus circumferentially with the umbilical ligament connected and then dropped into the abdominal cavity. The 12-mm camera port was placed in the umbilical hole. We prepared the five working ports (Figure 3(a)). Two were inserted at the height of the umbilicus on the bilateral middle clavicular line, and the other three were inserted at the inside of the right upper abdomen and outside of the bilateral lower abdomen. First, we inserted a camera into the right side working port of the umbilicus height and dissected the urachal ligament and adjacent tissues toward the bladder using the remaining right working port (Figure 3(b)). We transferred the camera to the central camera port and dissected the umbilical ligament until just above the bladder dome. We could confirm the bulging of the bladder diverticulum just to the right side of the bladder dome (Figure 3(c)). We used the cystoscopy to visualize the diverticular neck. The cystoscopic light could be observed from the abdominal cavity, and the bladder diverticulum was circumferentially resected at its neck. The resection line was extended to the bladder dome, and the urachal diverticulum was also resected (Figure 3(d)). The specimens were placed in an organ retrieval bag and removed through the 12-mm camera port site. The bladder defect was closed in 2 layers with a running 3-0 V-loc suture. The total operative time was 216 min with minimal blood loss.

The Foley catheter was removed on the seventh postoperative day, and she was discharged home on the same day. For 18 months after the operation, she was able to void without residual urine, urinary tract infection ceased to develop, and had no recurrence of the diverticula (Figure 1(f)). Microscopic hematuria also disappeared.

## 3. Discussion

Urachal remnants; that is a rare congenital anomaly, represent a failure in the obliteration of the allantois at birth that connects the bladder to the umbilicus. The most common symptoms and signs at diagnosis is umbilical discharge, while an incidental diagnosis is increasing as diagnostic imaging improves [1]. Spontaneous resolution without further intervention has been reported; however, it has also been suggested that chronic exposure to urinary stasis, infection, and inflammation in the remnant may predispose to urachal carcinoma. Therefore, there is no consensus on the management of Urachal remnants.

Bladder diverticula represent herniation of the urothelial lining that project through a congenital or acquired weakness in the muscular wall of the bladder [2]. Acquired bladder diverticula are associated with increased intravesical pressure secondary to bladder outlet obstruction but are often clinically insignificant and most of the bladder diverticula do not require intervention. On the other hand, surgical treatment of bladder diverticula is indicated in case of a tumor or stones in the diverticulum, persistent LUTS despite medications, recurrent UTIs, and a large amount of residual urine [2]. Several treatment strategies for bladder diverticula have been advocated, including surgical excision, and endoscopic management.

The patients with bladder diverticula were reported to have a higher risk for development of urothelial carcinoma, which can occur synchronously or precede carcinoma of the nondiverticular bladder [3]. A significantly higher percentage of diverticulum-associated bladder carcinomas are said to be high-grade and invasive compared with their non-diverticulum-associated counterparts because attenuation of the muscle layer associated with diverticulum formation may facilitate tumor invasion into peridiverticular soft tissues. Follow-up must be taken for the patients such as the present case.

Laparoscopic diverticulectomy is said to be technically feasible and safe and thought of as representing an alternative to the standard open procedure [4]. In the present case, the patient complained of intractable bladder diverticulitis and underwent laparoscopic bladder diverticulectomy and excision of urachal remnant simultaneously. Endoscopic fulguration of bladder diverticula was not recommended for this young patient because of the possibility of the remaining diverticulum and the risk of development urothelial carcinoma. The previous report suggested a laparoscopic approach for each of these diseases can be used safely, effectively, and minimally invasive.

## 4. Conclusion

We reported a patient who underwent laparoscopic bladder diverticulectomy and excision of urachal remnant simultaneously. Laparoscopic surgery was performed without any complications. Moreover, postoperative urination was good. Follow-up must be taken for the patients such as the present case because of a higher risk for the development of urothelial carcinoma.

## Abbreviations

LUTS: Lower urinary tract symptoms
UTIs: Urinary tract infections
CT: Computed tomography.
